# Integration of metabolic and transcriptomic analyses for revealing the galactose metabolism of tobacco (*Nicotiana tabacum*) under salt stress

**DOI:** 10.3389/fpls.2025.1614515

**Published:** 2025-06-17

**Authors:** Ge Bai, Hui Zhang, Yong Li, Da-Hai Yang, Mingliang Fei, Tao Pang, Yaning Fu, Ai-Guo Yang, Zhen-Yu Wang, Jinbao Gu, He Xie

**Affiliations:** ^1^ Key Laboratory of Tobacco Biotechnological Breeding, National Tobacco Genetic Engineering Research Center, Tobacco Breeding and Biotechnology Research Center, Yunnan Academy of Tobacco Agricultural Science, Kunming, Yunnan, China; ^2^ Application Department 1, Beijing Life Science Academy, Beijing, China; ^3^ National Tobacco Gene Research Centre, Zhengzhou Tobacco Research Institute, Zhengzhou, Henan, China; ^4^ Tobacco Research Institute, Chinese Academy of Agricultural Sciences, Qingdao, Shandong, China; ^5^ Institute of Nanfan and Seed Industry, Guangdong Academy of Sciences, Guangzhou, Guangdong, China

**Keywords:** salt stress, metabolomics, transcriptomics, tobacco plants, molecular mechanisms, salt tolerance

## Abstract

**Background:**

Soil salinization poses a global threat to agriculture, necessitating strategies to enhance plant salt stress tolerance. Understanding the metabolic and transcriptomic responses of tobacco plants to salt stress is crucial for developing such strategies.

**Results:**

This study identified 238 up-regulated and 122 down-regulated metabolites in tobacco plants under long-term salt stress. Initial stress stages activated galactose and sucrose metabolic pathways. Chlorophyll synthesis was impacted by decreased 5-aminolevulinic acid production, while proline accumulation helped mitigate cell damage. Metabolite-metabolite correlation analysis revealed significant correlations among metabolites, and enrichment analysis highlighted benzamides, amino acids, fatty acids, and monosaccharides. Transcriptome analysis identified 8,386 differentially expressed genes, with enriched pathways in hormone signaling, photosynthesis, and amino acid metabolism. Integrated analysis confirmed the involvement of sucrose pathway in the salt response, validated by qRT-PCR.

**Conclusions:**

This study provides a comprehensive understanding of the regulatory networks in tobacco during salt stress. The findings lay the groundwork for future research on plant stress responses and the development of salt-tolerant tobacco cultivars.

## Introduction

Soil salinity has a significant impact on seed germination, plant growth and development, as well as crop productivity worldwide. High salinity leads to the excessive accumulation of Na^+^ in plants, creating hyperosmotic and hyperionic conditions. These conditions hinder the uptake of water and nutrients from the soil (Yang and Guo, 2018). Plants have evolved a wide range of physiological and biochemical mechanisms to adapt to salt stress (Arif et al., 2020). These mechanisms include the regulation of ion and osmotic balance, as well as the management of stress-induced damage (Verslues et al., 2006). In response to salt stress, plants have been observed to over-accumulate various compatible solutes, including sugars, amino acids, and phospholipids (Shao et al., 2009). Additionally, various detoxification strategies have been developed to mitigate the detrimental effects induced by excessively stimulated reactive oxygen species (ROS) in the presence of high salinity (Choudhury et al., 2017; Yang et al., 2024). Moreover, it has been observed that a variety of stress-responsive genes, such as transporters, transcription factors, and protein kinases, exhibit differential expression patterns in response to salt stress (Zhu, 2016; Yang and Guo, 2018; Zhang et al., 2022). Therefore, the exploitation and utilization of saline land through the implementation of genetic engineering to enhance the salt tolerance of plant varieties may yield significant advantages in agricultural practice.

Metabolic profiles have been utilized to elucidate the role of plant adaptation to environmental stress (Urano et al., 2010). Several studies have indicated the involvement of primary metabolites, including sugars, amino acids, and organic compounds, in the response to salt stress (Kim et al., 2007; Li et al., 2021). Meantime, in response to salt stress, plants exhibit an increase in secondary metabolites such as phenols, saponins, flavonoids, carotenoids, polyamines, and lignin. These metabolites play a crucial role in plant protection through various functions, including scavenging reactive oxygen species (ROS), acting as antioxidants, and serving as regulatory molecules (Ashraf et al., 2018; Yang et al., 2024). Hence, the identification of crucial metabolites and the comprehensive analysis of plant metabolites not only elucidate the intricate mechanisms governing the interactions between plants and environmental stress, but also offer a viable strategy to enhance the selection of desirable phenotypes with improved salt tolerance. To date, various technological methods such as gas chromatography-mass spectrometry (GC-MS), liquid chromatography-mass spectrometry (LC-MS), nuclear magnetic resonance (NMR), and Fourier transform ion cyclotron resonance-mass spectrometry (FTICR-MS) have been utilized for the identification of plant metabolites (Barrett, 2012; Zhang et al., 2012). GC-MS has been increasingly applied in plant sciences to analyze a broad range of plant metabolites, particularly secondary metabolites (https://www.sciencedirect.com/science/article/abs/pii/S0165993622002783, https://revues.imist.ma/index.php/JASAB/article/view/40209). GC-MS is recognized for its ability to separate, identify, and quantify compounds such as volatile organic compounds (VOCs), fatty acids, and terpenes. These compounds play crucial roles in plant-environment interactions, including plant defense mechanisms, signaling, and adaptation to stress (https://onlinelibrary.wiley.com/doi/abs/10.1111/tpj.15176). For instance, GC-MS has been used in metabolomics to study plant responses to biotic and abiotic stressors, uncovering shifts in metabolic pathways that reflect physiological adaptations (https://www.sciencedirect.com/science/article/pii/S0048969722032521). Numerous studies have been conducted to characterize the metabolomic response to abiotic stress in various plant species. For instance, research has been carried out on tomato (Naik et al., 2023), soybean (Feng et al., 2020), barley (Wang et al., 2019), *Casuarina glauca* (Graca et al., 2019), *Aeluropus lagopoides* (Paidi et al., 2017), and wheat (Michaletti et al., 2018). These studies aim to understand the metabolic changes that occur in these plants under abiotic stress conditions. Additionally, nuclear magnetic resonance (NMR) spectroscopy was employed to investigate the alterations in metabolites within tobacco plants subjected to high salinity conditions. The results revealed that over 40 metabolites, such as organic acids, amino acids, carbohydrates, pyrimidines, and purines, exhibited significant changes (Zhang et al., 2011). Nevertheless, the application of NMR technology has resulted in the identification of a limited number of metabolites. In contrast, GC-MS has gained widespread usage due to its ability to identify secondary metabolites and its notable advantages in terms of high resolution, sensitivity, and reproducibility (Beale et al., 2018). In the present investigation, we conducted a comprehensive analysis by integrating metabolic and transcriptomic approaches to elucidate the metabolite profiling in tobacco plants subjected to prolonged salt treatment. We synthesized RNA-seq and metabolomics analysis and found that the sucrose pathway plays an important role in tobacco salt stress response. Furthermore, we aimed to identify the crucial metabolites that could serve as potential biomarkers for enhancing tobacco salt tolerance. Therefore, our study aims to provide novel insights into the metabolome process in tobacco in response to high salinity. This research could potentially be utilized in the breeding of salt-resistant tobacco cultivars, contributing to advancements in the field.

## Materials and methods

### Plant growth conditions and salt stress treatment

Seeds of the tobacco cultivar, Yunyan87, were obtained from the Yunnan Academy of Tobacco Agricultural Sciences (Yunnan, China). Seeds were subjected to surface sterilization by immersing them in a 10% (v/v) bleach solution including 0.1% tween 20 for a duration of 10 minutes. This was followed by three rinses in sterile distilled water. Subsequently, the sterilized seeds were directly sown into pots containing soil (1: 1 by volume of peat moss, vermiculite). The tobacco seedlings at an early stage of growth were cultivated in a controlled environment chamber with a photoperiod of 16 hours of light and 8 hours of darkness. The light source used was continuous white light (∼75 mol m^−2^ s^−1^) at 28°C-day/23°C-night. All plants were adequately irrigated following the process of sowing. Fifteen-day-old seedlings were exposed to a salt treatment using 300 mM NaCl for the specified duration (Song et al., 2019). Then the leaves were harvested and immediately frozen in liquid nitrogen for further processing. The quality control (QC) sample was generated by pooling equal amounts of all acquired samples.

### GC-MS sample preparation

The tobacco leaves, which had been recently frozen, were pulverized into a consistent powder using a CryoMill mill (Retsch, Haan, Germany) and subsequently stored at a temperature of -80°C until the commencement of the metabolic study. The leaf powder (100 mg) was introduced into a 2 mL Eppendorf tube. Subsequently, 450 μL of MTBE, 370 μL of methanol, and 680 μL of water were introduced into the tube and agitated for a duration of 2 minutes. The sample underwent vortexing for a duration of 5 minutes in order to extract the metabolites, followed by centrifugation at 18,900 g for a period of 10 minutes. The substratum (600 μL) was subjected to freeze-drying in Labconco vacuum concentrators for a duration of 6 hours. In preparation for GC-MS analysis, the resulting dry residue was combined with 100 μL of a methoxyamine solution (20 mg/mL in pyridine) and incubated in a culture room at 37°C for a period of 90 minutes. This step was carried out to facilitate the oximation reaction, which serves to protect the carbonyl group and reduce the occurrence of isomers. The silylation process was carried out by introducing 80 μL of MSTFA to the sample and allowing it to incubate for 30 minutes in a culture room maintained at a temperature of 37°C. The samples were subsequently subjected to centrifugation for a duration of 5 minutes. Following centrifugation, the resulting supernatant was carefully transferred into a conical insert located within a 2-mL glass vial. This transfer was performed in preparation for subsequent analysis using GC-MS.

### GC-MS measurement

The sample that had undergone derivatization was introduced into an Agilent 7890A Gas Chromatograph (GC) coupled with an Agilent 5977B Mass Spectrometer (MS) (Agilent Technologies, DE, USA). The GC separation was conducted using an Agilent DB-5 MS fused silica capillary column (30 m × 0.25 mm × 0.25 μm). The temperature of the GC oven was initially set at 60°C for the first minute and subsequently raised at 5°C/min to 280°C for a duration of 15 minutes. The injection temperature was set at 280°C, and the injection volume was 1 μL with a split ratio of 20:1. Helium, with a purity of 99.9995%, was utilized as the carrier gas. A constant flow rate of 1.0 mL/min was employed for the separation process. The MS was configured to operate in full-scan mode, covering a mass range of 33−600 m/z, with a scan speed of 5 scans per second. The solvent delay time was set to 6 minutes. The temperatures of the interface and the ion source were adjusted to 280°C and 240°C, respectively. In order to achieve ionization of the metabolites, the electron impact (EI) model with an energy of 70 eV was employed. The voltage of the detector was consistently maintained at 1.2 kV.

### Ultra-performance liquid chromatography coupled with quadrupole time-of-flight mass spectrometry analysis

An experimental setup consisting of a Waters ACQUITY UPLC system (Waters Corporation, MA, USA) coupled with a Bruker Impact II ESI-Q-TOF-MS (Bruker Corporation, USA) was employed to obtain precise parent/product mass spectra of the extracted metabolites. The experimental conditions included the use of a separation column, specifically the Waters BEH C18 column (100 × 2.1 mm id, 1.7 μm particle sizes, Waters Corporation). The temperature of the column was adjusted to 30°C. The composition of the mobile phase included water with 0.1% formic acid (referred to as mobile phase A) and acetonitrile (referred to as mobile phase B). The gradient elution was performed using a flow rate of 0.28 mL/min. Initially, the mobile phase composition was set at 10% B for 1 minute. Subsequently, the proportion of solvent B was linearly increased from 1 to 18 minutes, reaching 90% B. This composition was maintained for 3 minutes. At 21.01 minutes, the mobile phase composition was switched back to 10% B and maintained for an additional 3 minutes. The volume of the injection was 5 μL. Samples were injected randomly in order to mitigate potential systematic errors. In addition to the inclusion of samples of interest, the experiment process involved the systematic addition of quality control (QC) samples at regular intervals.

The MS analysis was conducted using a Bruker Impact II ESI-Q-TOF-MS (Bruker Corporation, USA). Data were stored in centroid format and were collected within the mass range of 80 to 1200 Da. The conditions for the MS analysis were as follows: the flow rate of the nitrogen drying gas was set at 8 L/min, the gas temperature was maintained at 200°C, the pressure of the nitrogen nebulizing gas was set to 29 psi, and the capillary voltage was set at 4500 V. Prior to each acquisition, calibration of the mass axis was performed using a sodium formate solution. Auto MS/MS was employed for identification, utilizing a dynamic collision energy range of 15–50 V.

### Data analysis of the metabolome

Mass calibration was conducted using the Bruker Data Analysis software. The raw data, which had been calibrated, was subsequently processed using MS-Dial 4.6 software. After undergoing the procedures of noise removal, extraction of molecular features, alignment of peaks, and annotation of features, a peak table containing both quantitative and qualitative information of the identified metabolites was obtained. Differentially accumulated metabolites were identified using a statistical significance threshold of P < 0.05. The software MetaboAnalyst 6.0 was employed for conducting pathway analysis, statistical comparison, and visualization (https://www.metaboanalyst.ca/) (Pang et al., 2021).

### RNA-seq library preparation and data analysis

Aliquots derived from an identical tissue pool were allocated to parallel processing for RNA extraction and metabolite profiling. Three biological replicate samples were utilized for the purpose of RNA extraction. Total RNA was extracted from plants utilizing TRIzol reagent (Invitrogen Corp., Carlsbad, CA). RNA purifications were conducted utilizing the RNeasy Mini Kit (Qiagen, Chatsworth, CA). Library preparation was conducted following the protocol provided by the Illumina Hiseq RNA sample preparation kit (Illumina, San Diego, CA). The RNA-sequencing analysis was conducted by Novogene (Beijing, China). FeatureCounts v1.5.0-p3 was used to count the reads numbers mapped to each gene. Differential expression analysis of two conditions/groups (two biological replicates per condition) was performed using the DESeq2 R package (1.16.1). The GO enrichment and KEGG analysis for the differentially expressed genes were obtained using clusterProfiler (version 3.8.1). The correction of the deviation from gene length was implemented. For those genes with corrected P value of less than 0.05, GO enrichment were also analyzed.

### Quantitative real-time PCR analysis

2 μg of total RNA in a 20 μl reaction was converted to cDNA with a SuperScript III Reverse Transcriptase (Invitrogen, USA) by manufacturer instructions on a Eppendorf Mastercycler thermocycler (Eppendorf AG, Germany) with the following conditions: 25°C for 5 min, 50°C for 60 min, 70°C for 15 minutes, followed by a hold at 4°C until use in RT-qPCR reaction. 60 μl deionized water was added into 20 μl cDNA, and 1 μl of diluted cDNA mixture was used as the input for qPCR reaction. qPCR reactions were made with a SuperReal PreMix Plus SYBR Green Kit (TIANGEN Biotech, China), following manufacturer instructions in a 20 μl volume. qPCR was done on an Applied Biosystems™ QuantStudio™ 6 Flex Real-Time PCR System (ThemoFisher Scientific, USA) with the following cycling conditions: 95°C for 15 min, followed by 40 cycles of 95°C for 10 sec, 60°C for 20 sec, and 72°C for 32 sec. Melt curve conditions were 95°C for 15 sec, 60°C for 1 min, 95°C for 15 sec. All samples had only one melt temperature peak. Log_2_fold change was calculated by the 2^-ΔΔCT^ method using 26S as a reference gene (accession number NC_006581.1). CT values represent the average of three technical replicates. Sequences of primers used for RT-qPCR are listed in **Supplementary Table S4**.

## Results

### Assigning metabolites in tobacco plants in response to salinity

**Figure 1:**
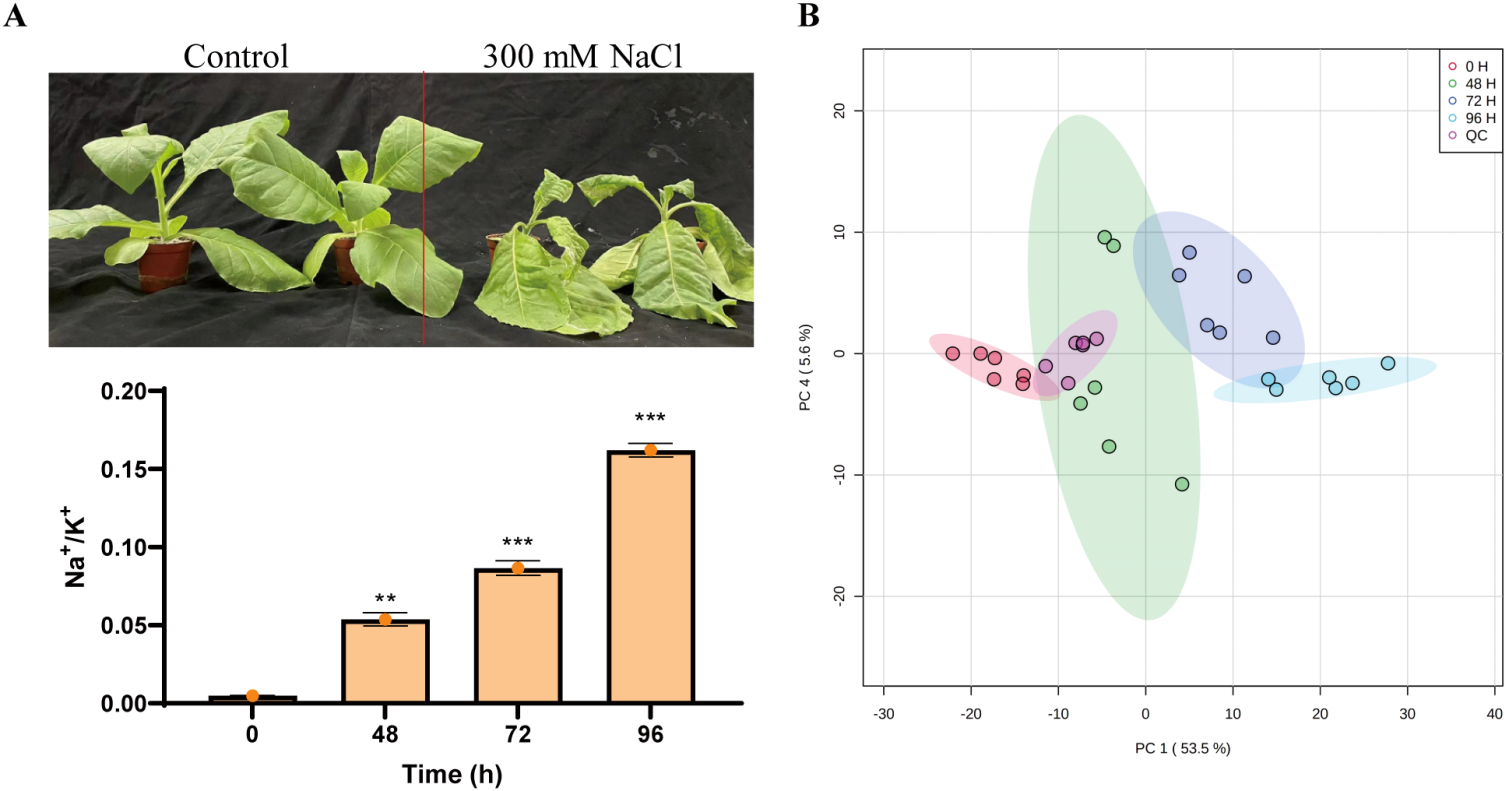
Assigning metabolites in tobacco plants in response to salinity. **(A)** The phenotype of tobacco plants in response to salt. Fifteen-day-old seedlings were exposed to a salt treatment using 300 mM NaCl for five days. **(B)** Principal component analysis (PCA) of samples subjected to metabolism analysis.

Maintaining cellular Na^+^/K^+^ homeostasis is essential for plant growth under salt stress. Consistent with previous study (Selvakumar et al., 2018), salt stress caused the reduction of ratio of K^+^ and Na^+^ in tobacco plants that resulted in wilt phenotype (**Figure 1A**), suggesting that tobaccos have been seriously affected by high salinity. To evaluate the effects of metabolic homeostasis in tobaccos responding to salt stress, the metabolite profiles of the tobacco plants were subjected to 300 mM NaCl for indicated time. Principal component analysis (PCA) has been demonstrated that all samples were distributed within the 95% confidence range. There were 54.4% and 6% metabolites in the PC1 direction and the PC4 dimension, respectively. The score plot displayed four different groups, which were associated with different salt-treated samples at indicated sampling time (**Figure 1B**), indicating a distinct separation of the metabolite profiles under salt stress conditions.

To investigate the metabolic profiling of tobacco in response to salt using high-resolution mass spectrometry (HRMS), we utilized the MetaboAnalyst webtool (version 5.0) with automated parameter settings. The differentially accumulated metabolites (DAMs) were identified among four treatments with using a statistical significance threshold of p < 0.05. (**Supplementary Table S1**). It was shown that 238 metabolites were up-regulated, and 122 metabolites were down-regulated under salt stress condition (**Supplementary Table S1**). There were 46 and 181 metabolites whose contents were increased at 48 h and 72 h after salt treatment, respectively, and the contents of 19 metabolites were significantly increased at 96 h after salt treatment (**Supplementary Table S1**). Additionally, it was observed that the levels of metabolites decreased at 48 h and 72 h after salt treatment, with 42 and 26 metabolites exhibiting lower contents, respectively (**Supplementary Table S1**). Meantime, 35 metabolites which showed the highest contents at 48 hours were decreased under prolonged salt treatment, and 19 metabolites showed the highest contents at 72 hours but decreased under prolonged salt treatment (**Supplementary Table S1**).

### Metabolite-metabolite correlation analysis

**Figure 2:**
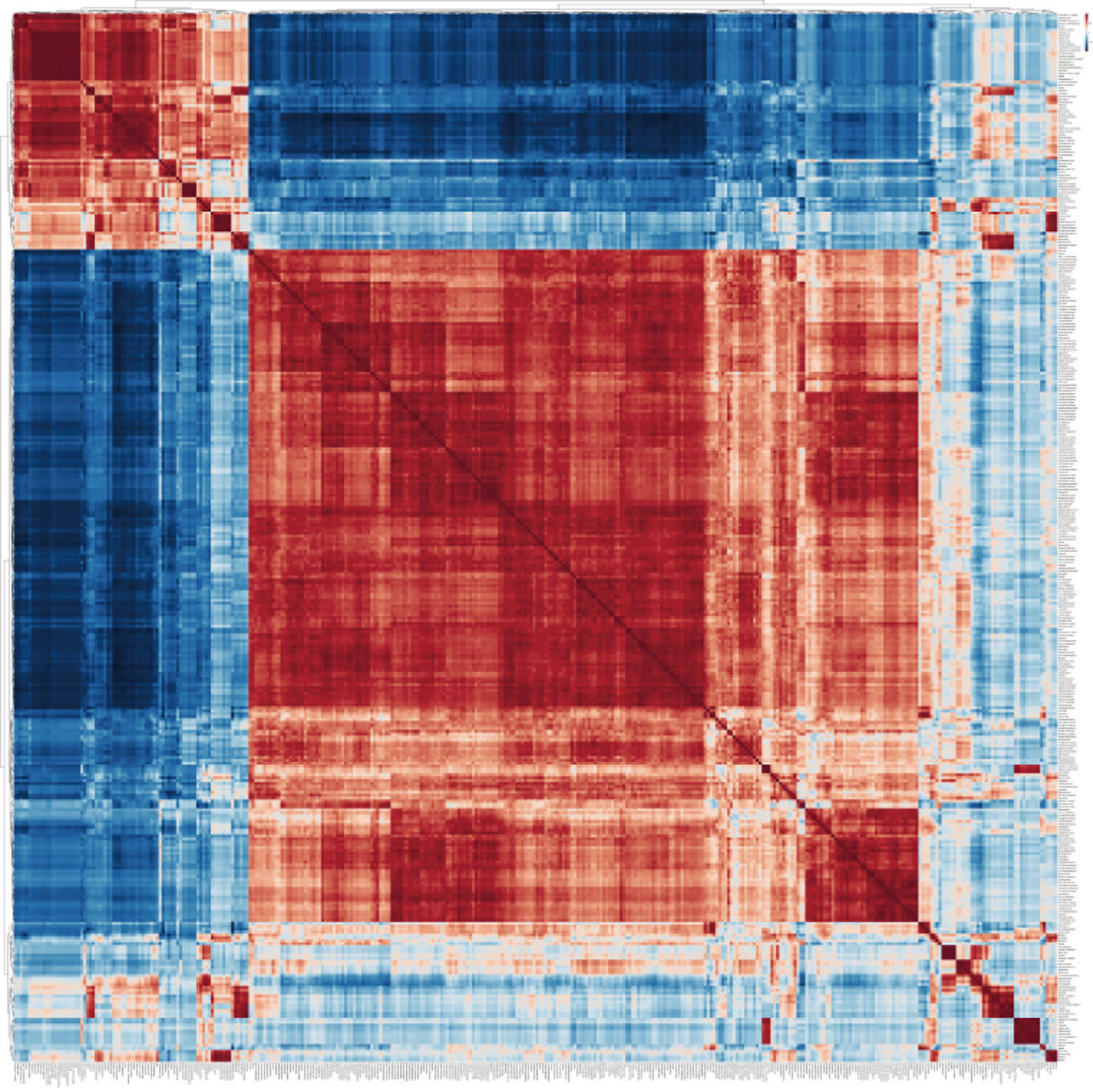
Metabolite-metabolite correlation analysis of tobacco plants in response to salinity. The software MetaboAnalyst 6.0 (https://dev.metaboanalyst.ca/ModuleView.xhtml) was employed for conducting metabolite-metabolite correlation analysis.

It was found that most metabolites in tobacco showed positive correlation after salt stress and among them, 277 metabolites significantly showed positive correlation (**Figure 2**). In addition, a total of 66 metabolites exhibited a significant negative correlation with the majority of other metabolites (**Supplementary Table S2**), while some metabolites displayed a neutral correlation. The metabolites with positive correlation were mainly belong to benzamides, amino acids and peptides, fatty acids and conjugates, and monosaccharides subcategories, including 14 benzamides, 12 amino acids and peptides, 5 fatty acids and conjugates, 5 monosaccharides (**Supplementary Table S2**). The subcategories of amino acids and peptides included carnosine, betaine, glycine, L-tyrosine, L-phenylalanine, L-proline, L-leucine, homocarnosine, N-acetylleucine, L-histidine, and trimethylbetaine. The subcategories of monosaccharides included glycerol, glyceric acid, D-ribose, mannitol, and ribonic acid (**Supplementary Table S2**). Among the 66 metabolites with negative correlation, most of them were amino acids and peptides, fatty acids and conjugates and monosaccharides (**Supplementary Table S2**). The amino acids and peptide consisted of L-glutamic acid, L-phenylalanine, L-alanine, L-threonine, L-isoleucine, L-lysine, L-glutamine, L-methionine, and L-valine. The fatty acids and conjugates contained gamma-aminobutyric acid, adipic acid, stearic acid, succinic acid, methylmalonic acid. The monosaccharides that were included in the study were sorbitol, gluconic acid, threonic acid, and pectin.

### Differential metabolites of tobacco in response to salt

**Figure 3:**
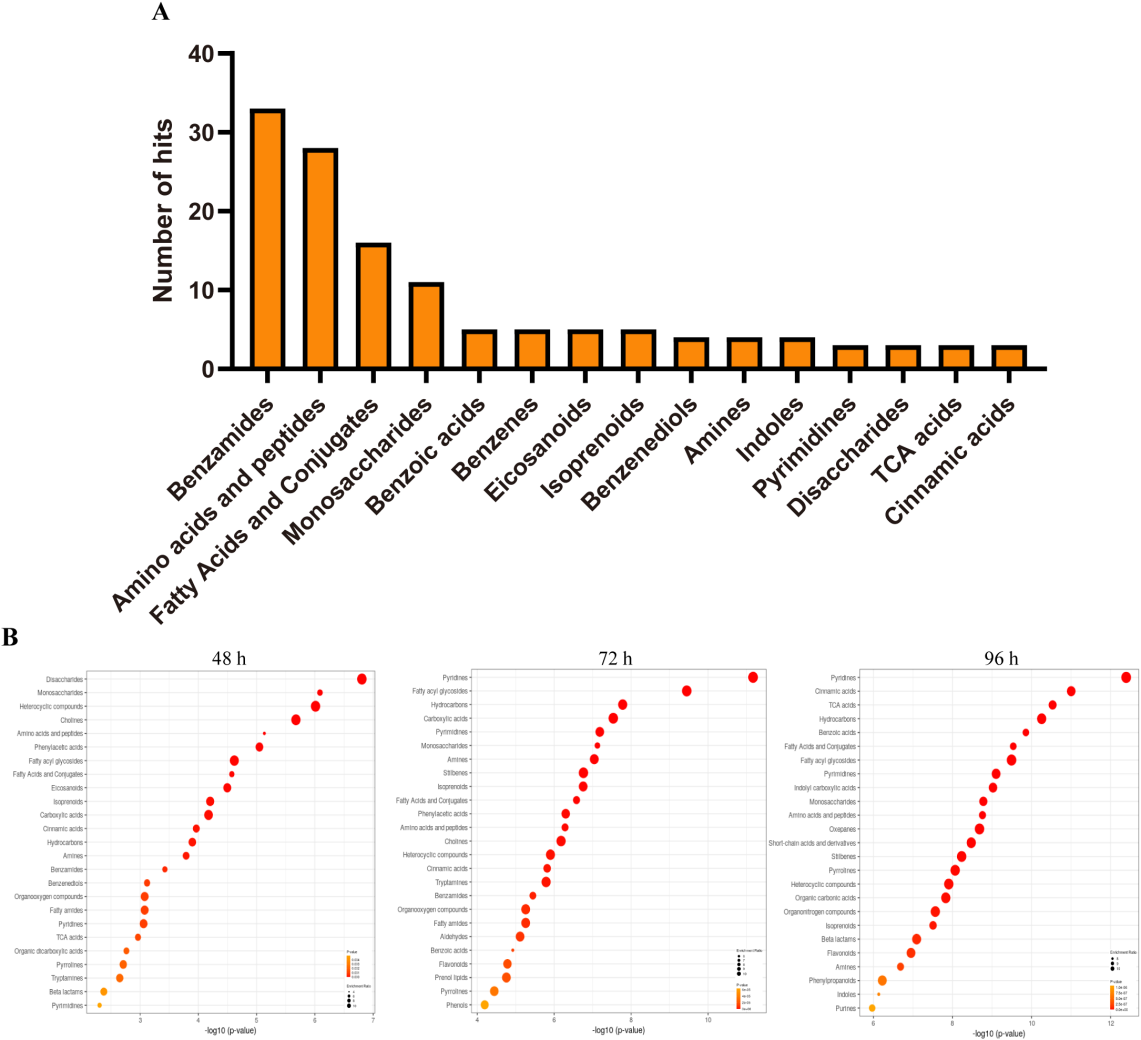
Differential metabolites of tobacco plants in response to salinity. **(A)** The number of metabolite enrichment in tobacco responds to salt stress. **(B)** The overview top 25 of enriched metabolites sets in tobacco responds to salt stress at indicated time.

To gain a deeper understanding of the metabolite in tobacco under conditions of salt stress, an enrichment analysis of the metabolite was conducted. The analysis revealed that the initial four categories of enrichment were classified as benzamides, amino acids and peptides, fatty acids and conjugates, and monosaccharides. These categories consisted of 33, 28, 16, and 11 metabolites, respectively (**Figure 3A**). The remaining enrichments of metabolites are found within a set of 10 metabolites. The variations in the number of metabolites and the changes in their content in each enrichment suggest that the number of metabolites could potentially indicate the importance or significance of these metabolites. Consequently, an additional analysis of metabolic enrichment was conducted. We conducted a comparative analysis of the samples at 48 h, 72 h, and 96 h in relation to the sample at 0 h, respectively. The results showed that significant changes were observed in monosaccharides, amino acids and peptides, and fatty acyl glycosides after 48, 72, and 96 hours of salt treatment, indicating that these three metabolites may play important roles in salt stress response. In addition, significant differences were observed in fatty acids and conjugates after 48 and 72 hours of salt treatment, and significant differences were observed in pyridine and pyrimidine after 72 and 96 hours of treatment (**Figure 3B**).

**Figure 4:**
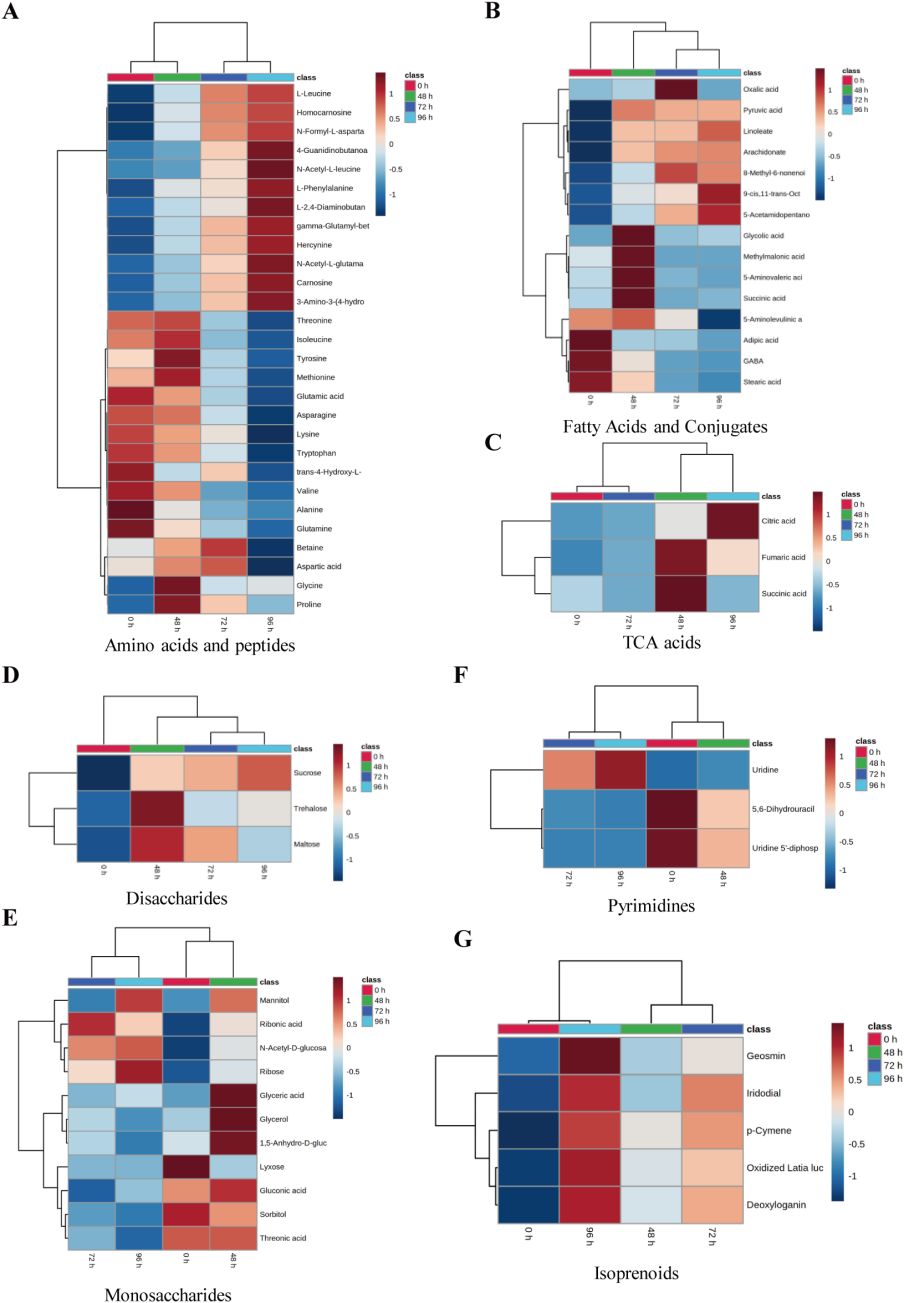
The clustering analysis of significance enriched metabolites of tobacco under salt treatment. The heatmap showed the results of the clustering analysis for amino acids and peptides **(A)**, fatty acids and conjugates **(B)**, TAC acids **(C)**, disaccharides **(D)**, monosaccharides **(E)**, pyrimidines **(F)**, and isoprenoids **(G)**. Rows and columns represent metabolites and treatment groups, respectively. The red color indicates a high abundance of a metabolite, whereas the blue color represents a low relative abundance of a metabolite.

To comprehensively examine the alterations in tobacco metabolites following exposure to salt stress, the primary metabolites were subsequently extracted for the purpose of constructing the heatmap. After salt treatment, 12 metabolites in amino acids and peptides were significantly downregulated, 12 metabolites were significantly upregulated, and 4 metabolites exhibited an initial increase followed by a subsequent decrease during prolonged exposure to salinity (**Figure 4A**). Among fatty acids and conjugates, six metabolites were significantly upregulated, three metabolites were significantly downregulated, and five metabolites first increased and then decreased after salt treatment (**Figure 4B**). Interestingly, the levels of metabolites associated with tricarboxylic acid (TCA) cycle acids and disaccharides exhibited a discernible pattern as the duration of salt stress increased. The concentration of citric acid reached its peak at 72 hours after salt treatment, whereas the concentrations of fumaric acid and succinic acid were highest at 48 hours and subsequently decreased at 72 hours (**Figure 4C**).

Among the metabolites of disaccharides, the sucrose content exhibited a gradual increase, whereas trehalose and maltose displayed the highest levels at 48 hours. Subsequently, these metabolites experienced a decrease at 72 hours following the salt stress treatment (**Figure 4D**). The levels of glyceric acid, glycerol, 1,5-Anhydro-D-glucitol, and monosaccharide metabolites exhibited a peak at 48 hours following salt treatment, followed by a subsequent decrease. The concentrations of gluconic acid, sorbitol, and threonic acid exhibited a significant decrease 72 hours following salt treatment (**Figure 4E**). The levels of mannitol, n-acetyl-D-glucosamine, ribose, and lyxose were found to increase with the duration of salt treatment. In contrast, ribonic acid exhibited the highest concentration at the beginning of the experiment (0 h) but subsequently decreased (**Figure 4E**). Among the pyrimidines, the levels of uridine 5’-diphospho-N-acetylglucosamine and 5,6-dihydrouracil exhibited a gradual decrease following salt treatment. However, the concentration of uridine did not show an increase until 72 hours after salt treatment (**Figure 4F**). After subjecting the tobacco plants to salt treatment, the levels of five isoprenoids exhibited an increase over time as the stress was prolonged (**Figure 4G**). Metabolites such as citric acid, sucrose, trehalose, and glycerol can enhance plant salt tolerance by increasing photosynthetic rate, reducing reactive oxygen species, and regulating osmotic pressure (Bahieldin et al., 2013; Tahjib-Ul-Arif et al., 2021; Ibrahim, 2025; Sevgi and Leblebici, 2025).

### Metabolic pathway analysis of differential metabolites in tobacco under salt stress

**Figure 5:**
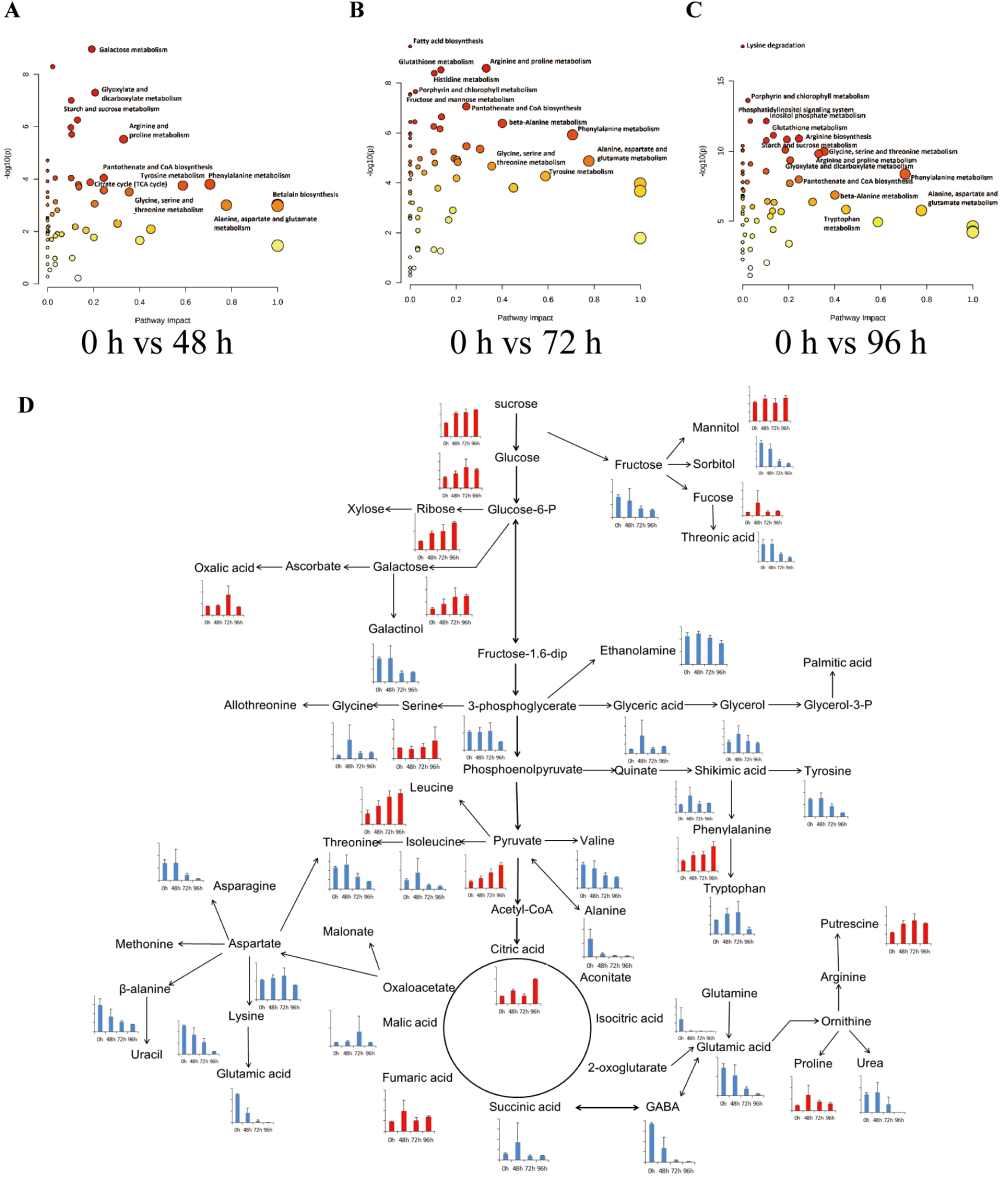
Metabolic pathway analysis of differential metabolites in the tobacco plants in response to salinity. The pathway impact analysis of tobacco plants responds to salt stress in 0 h vs 48 h **(A)**, 0 h vs 72 h **(B)**, and 0 h vs 96 h **(C)**. Each bubble in the bubble graph represents a metabolic pathway. The abscissa and size of the bubble represent the influencing factor of this pathway in the topological analysis; the larger the size is the greater impact factor. The ordinate and color represent the P-value of the enrichment analysis (− ln[P-value]); the deeper the color is, the smaller the P-value and the more significant the degree of enrichment. **(D)** The represented pathway related to sugar metabolism in tobacco responds to salt stress. The histograms showed the contents of differentially accumulated metabolites from the metabolome results.

To further explore the impact of salt stress on metabolites in tobacco, we conducted an analysis to examine the enrichment of metabolic pathways. This analysis involved comparing the metabolite contents at 48 h, 72 h, and 96 h with the initial contents at 0 h (**Figures 5A–C**). The findings indicated that several pathways were significantly enriched following a 48-hour salt stress treatment. These pathways included galactose metabolism, porphyrin and chlorophyll metabolism, glyoxylate and dicarboxylate metabolism, starch and sucrose metabolism, glycerophospholipid metabolism, phenylalanine, tyrosine and tryptophan biosynthesis, histidine metabolism, arginine and proline metabolism, as well as tropane, piperidine, and pyridine alkaloid biosynthesis (**Figures 5A–C**). Additionally, After subjecting the samples to a 48-hour salt treatment, the tobacco samples exhibited significant enrichment in the pathways related to pantothenate and CoA biosynthesis, Inositol phosphate metabolism, phosphatidylinositol signaling system, and citrate cycle (**Figure 5A**). After subjecting the samples to a 72-hour salt treatment, the pathways that exhibited significant enrichment were fatty acid biosynthesis, arginine and proline metabolism, glutathione metabolism, histidine metabolism, porphyrin and chlorophyll metabolism, pentose phosphate pathway, fructose and mannose metabolism, pantothenate and CoA biosynthesis, butanoate metabolism, nicotinate and nicotinamide metabolism, and amino sugar and nucleotide sugar metabolism (**Figure 5B**). After subjecting the samples to a 96-hour salt treatment, significant alterations were observed in various metabolic pathways. The pathways that exhibited the most pronounced changes included lysine degradation, porphyrin and chlorophyll metabolism, inositol phosphate metabolism, phosphatidylinositol signaling system, glutathione metabolism, arginine biosynthesis, galactose metabolism, starch and sucrose metabolism, lysine biosynthesis, pentose and glucuronate interconversions, TCA cycle, and fatty acid biosynthesis (**Figure 5C**). It was noted that sugar metabolism was involved in the enrichment of tobacco plants after exposure to salt stress, and several sugar-related metabolic were differentiated changed (**Figure 5D**). Therefore, the findings of this study indicate that the pathways involved in the enrichment of tobacco plants after exposure to salt stress primarily include sugar metabolism and other metabolisms amino acid metabolism, phospholipid, and chlorophyll metabolism.

### Transcriptome analysis of differentially expressed genes in tobacco under salt stress

**Figure 6:**
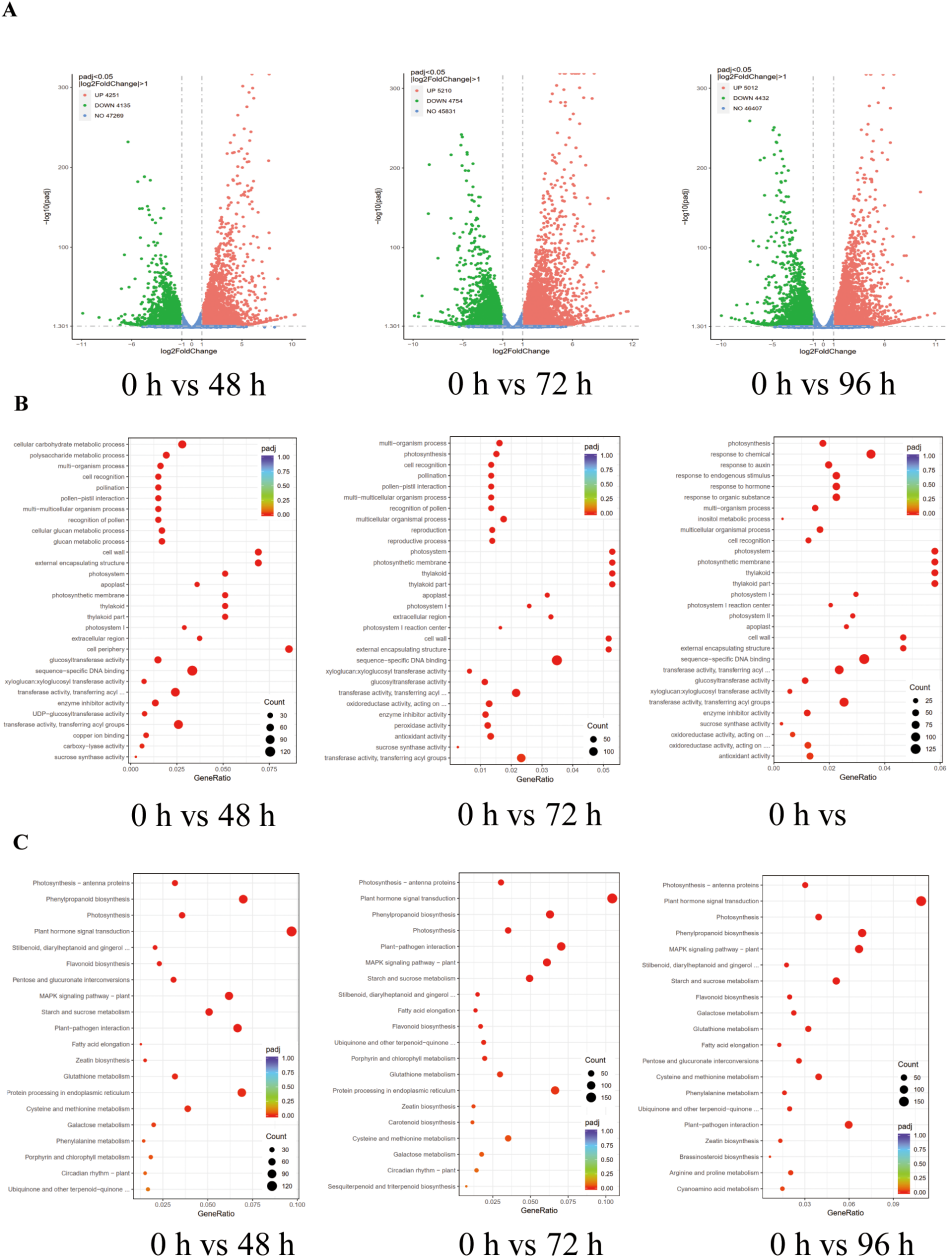
Transcriptome analysis of differentially expressed genes (DEGs) in tobacco under salt stress conditions. **(A)** Volcano plots analysis of DEGs in tobacco under salt stress conditions in 0 h vs 48 h, 0 h vs 72 h, and 0 h vs 96 h. **(B)** Gene ontology (GO) analysis of DEGS in tobacco under salt stress conditions in 0 h vs 48 h, 0 h vs 72 h, and 0 h vs 96 h. **(C)** Kyoto Encyclopedia of Genes and Genomes (KEGG) analysis of DEGS in tobacco under salt stress conditions in 0 h vs 48 h, 0 h vs 72 h, and 0 h vs 96 h.

To gain a comprehensive understanding of the global changes in differentially expressed genes in tobacco in response to salt stress, we conducted a systematic analysis of the tobacco transcriptome under salt stress conditions for a specified duration. Transcriptome profiling analysis demonstrated a significant number of DEGs in salt-treated plants at 48 h, 72 h, and 96 h compared to control plants (0 h). The DEGs were identified based on a fold change of ≥ 2 and a false discovery rate (FDR) of ≤ 0.05 (**Figure 6A**; **Supplementary Table S3**). The results of the study revealed that a total of 8,386 DEGs (4,251 up-regulated and 4,135 down-regulated) were detected after 48 hours of salt treatment, with 4,251 genes up-regulated and 4,135 genes down-regulated. Similarly, after 72 hours of salt treatment, 9,964 DEGs were identified, with 5,210 genes up-regulated and 4,754 genes down-regulated. Furthermore, after 96 hours of salt treatment, 9,444 DEGs were observed, with 5,012 genes up-regulated and 4,432 genes down-regulated. These findings were compared to the control plants at 0 hours (**Figure 6A**). These findings indicate that elevated salinity levels induce differential gene expression in tobacco. To gain a deeper understanding of the roles played by the DEGs in response to salt stress, a gene ontology (GO) analysis was conducted on these DEGs. It was found that several functional categories were over-represented in their comparison groups, e.g., ‘cell recognition’, ‘photosystem’, ‘sucrose synthase activity’, ‘sequence-specific DNA binding’, and antioxidant activity (**Figure 6B**). Subsequently, the Kyoto Encyclopedia of Genes and Genomes (KEGG) analysis was employed to identify the biological pathways of DEGs associated with salt stress. This analysis was conducted using the Database for Annotation, Visualization, and Integrated Discovery (DAVID) with a cut-off probability of ≥ 0.95 and a fold change of ≥ 2. It was noted that the pathways of ‘Plant hormone signal transduction’, ‘Photosynthesis - antenna proteins’, ‘Porphyrin and chlorophyll metabolism’, ‘Starch and sucrose metabolism’, ‘Arginine and proline metabolism’, ‘Phenylalanine’, ‘tyrosine and tryptophan biosynthesis, and Glycine’, and ‘serine and threonine metabolism’ were significantly changed in tobacco in response to salt stress (**Figure 6C**).

### Integrated transcriptomic and metabolomic analysis

**Figure 7:**
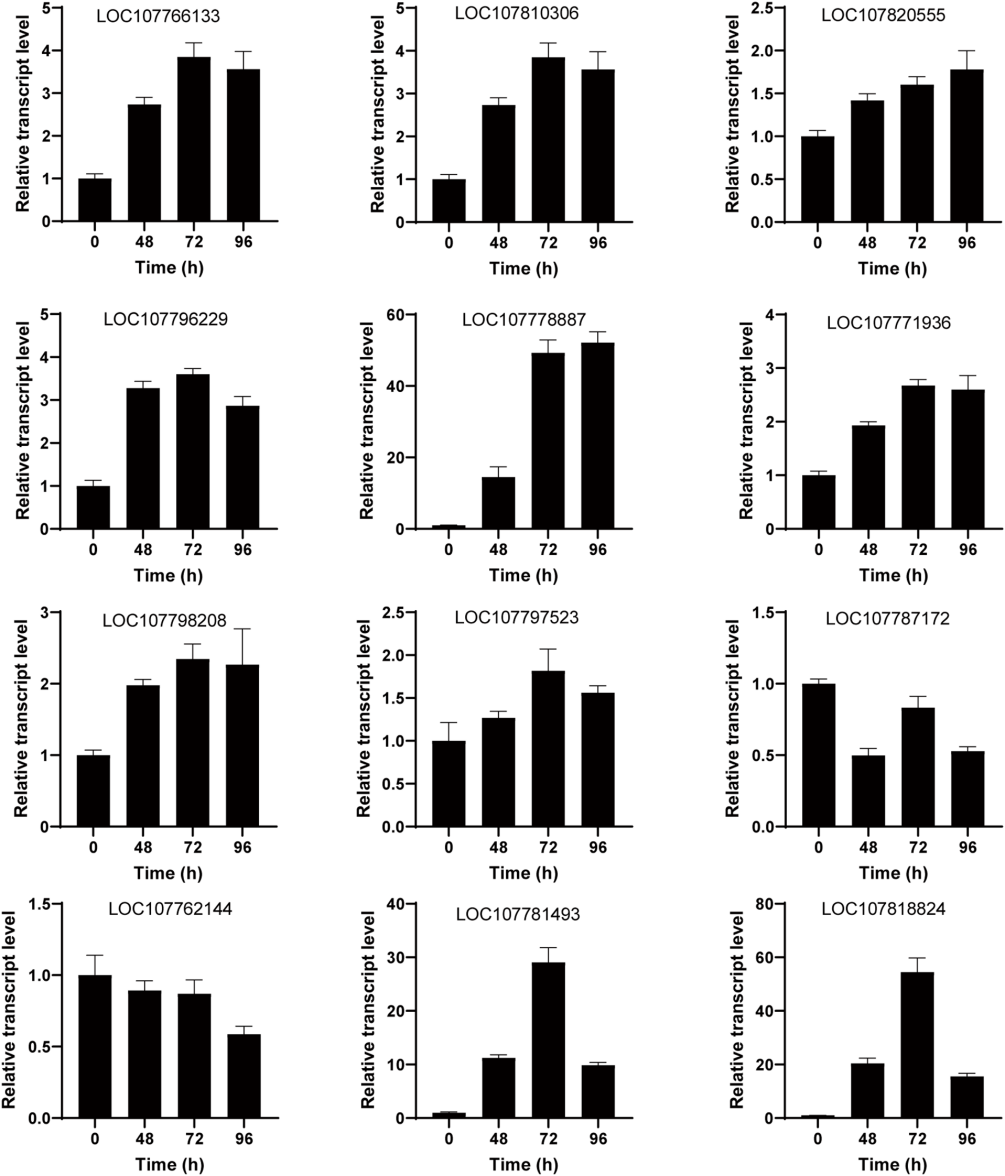
Validation of galactose metabolism, starch and sucrose metabolism-related genes using quantitative real-time polymerase chain reaction (qRT-PCR). The gene expression levels were measured under salt treatment at 0 h, 48 h, 72 h, and 96 h. The vertical bar represents the mean plus standard deviation, which was determined based on three replicates. *26S* was used as an internal control.

A comprehensive investigation was conducted to enhance the understanding of the regulatory network associated with salt stress response in tobacco. This investigation employed integrated transcriptomic and metabolomic analyses to provide a thorough examination of the subject matter. Analysis between DEGs and DAMs showed that the galactose metabolism, starch and sucrose metabolism were well enriched, suggesting that these metabolism pathways play an important role in response to salt stress in tobacco. To enhance the credibility of the transcriptomic findings and validate the dynamic expression of DEGs under salt stress conditions, a quantitative real-time polymerase chain reaction (qRT-PCR) was performed. The DEGs investigated in this study included *sucrose-phosphate synthase* (*SPS*, *LOC107766133*), *alpha-1,4 glucan phosphorylase L-1 isozyme* (*LOC107810306*), *endoglucanase 25-like* (*LOC107820555*), *sucrose synthase* (*LOC107796229*), *beta-glucosidase 11-like* (*LOC107778887*), *isoamylase 3* (*LOC107771936*), *4-alpha-glucanotransferase DPE2* (*LOC107798208*), *hexokinase-1* (*LOC107797523*), *aldose 1-epimerase-like* (*LOC107787172*), *beta-fructofuranosidase, insoluble isoenzyme CWINV1-like* (*LOC107762144*), *galactinol–sucrose galactosyltransferase 2;1* (*LOC107781493*), and *galactinol–sucrose galactosyltransferase 2;1* (*LOC107818824*). qRT-PCR results were consistent with transcriptomic data (**Figure 7**). SPS is a key rate-limiting enzyme in sucrose synthesis. After salt stress treatment, the expression level of *SPS* significantly increased, which is consistent with the increase in sucrose content after salt treatment.

## Discussion

Soil salinization is increasingly severe, and in the early stage, the response of tobacco to salt stress was mainly studied at the transcriptional level, exploring new salt tolerant or salt sensitive genes, with relatively little research on the metabolome. Therefore, the objective of this work is to evaluate the alterations in the metabolome of tobacco plants when exposed to salt stress conditions by employing GC-MS analysis. This technique may elucidate the metabolic regulatory processes implicated in the plant’s salt stress response in tobacco. In this study, we have successfully constructed a comprehensive network of metabolites in tobacco plants in response to salt stress treatment. This network represents the most extensive and detailed metabolite network reported thus far for tobacco under salt stress conditions (**Figure 2**). By analyzing the changes in metabolite levels, we have gained valuable insights into the metabolic responses of tobacco plants to salt stress. The present study demonstrates that photosynthesis serves as the primary source of energy during daytime, while metabolites such as starch and amino acids play a crucial role in providing energy during nighttime. This energy supply during nighttime is facilitated through various metabolic pathways including glycolysis, amino acid metabolism, and the tricarboxylic acid (TCA) cycle. These findings highlight the significance of these metabolic processes in maintaining energy homeostasis under normal conditions. The rates of photosynthesis and respiration in plants are commonly observed to decrease under stressful conditions, leading to a subsequent reduction in plant growth. The present study investigated the impact of salt stress on the content of 5-aminolevulinic acid (ALA) in tobacco plants. Our findings revealed a significant down-regulation of ALA content as the duration of salt stress increased (**Figures 2**, **4A**). The conversion of 5-aminolevulinic acid to porphobilinogen is a crucial step in the biosynthesis of chlorophyll. This reaction is catalyzed by an enzyme, as indicated by previous studies (Hotta et al., 1997). Ultimately, the production of the pyrrole ring, an essential component of chlorophyll, is facilitated through this enzymatic process. Hence, the findings of our study suggest that the reduction in photosynthesis observed in tobacco plants subjected to salt stress may be attributed to a decrease in the levels of 5-aminolevulinic acid (ALA), which subsequently leads to a decline in chlorophyll synthesis due to insufficient pyrrole ring formation.

Plants must change their transcription levels and metabolites in order to adapt to this energy deficiency, and amino acids play a critical part in this process (Baena-Gonzalez and Sheen, 2008). Tobacco has five distinct categories of identified amino acids. The alanine variants consist of alanine, valine, and leucine, which derive their carbon from pyruvate. The serine type (serine, glycine, and L-cysteine) and the glutamate type (glutamate, L-glutamine, and proline) are derived from α-ketoglutaric acid, an intermediate compound in the tricarboxylic acid cycle. The aspartic acid group comprises L-aspartic acid, L-asparagine, lysine, threonine, and L-isoleucine. These amino acids derive their carbon atoms from either oxalacetic acid or fumarate, which are intermediate products in the tricarboxylic acid cycle. The histidine and aromatic amino acids consist of histidine, tyrosine, tryptophan, and L-phenylalanine. Research has demonstrated that the presence of free amino acids can provide protection to plants against the harmful effects of stress (Hare and Cress, 1997; Yang et al., 2017). The levels of free amino acids in tobacco were increased 48 hours after salt treatment, a phenomenon that has been reported in several species including *Nitraria sibirica Pall*, *Porphyra haitanensis* (Fu et al., 2021), *Arabidopsis thaliana* (Huang and Jander, 2017), *Thellungiella salsuginea* (Lugan et al., 2010), and maize (Li et al., 2021). When plants are subjected to salt stress, it leads to the generation of osmotic stress, followed by ionic toxicity. This causes an excessive buildup of reactive oxygen species (ROS) and high levels of ammonia in plant cells (Martinelli et al., 2007). To prevent the harmful effects of elevated ammonia levels, plants typically convert ammonium ions into transaminergic metabolites (Skopelitis et al., 2006). Hence, augmenting the amino acid levels in tobacco can mitigate its cytotoxic effects. Proline has a crucial role in regulating cell osmotic pressure, safeguarding the integrity of the cell membrane, and eliminating reactive oxygen species (ROS). Additionally, it significantly improves plant resistance to drought and salt stress (Hare and Cress, 1997). Our findings revealed that the proline content doubled in the type of glutamic acids, while the levels of glutamic acid, glutamine, and GAGB decreased by more than five times after 48 hours of salt treatment (**Figure 4**). Glutamic acid and glutamine can be transformed into proline by 1-pyroline-5-carboxylate synthase (P5CS), leading to increased proline levels in order to counteract the negative effects of salt stress and prevent harm caused by excessive salt ions.

High-throughput transcriptome and metabolome profiling techniques allow us to comprehensively assess the changes in gene expression and metabolic product levels in tobacco plants subjected to salt stress. By integrating the transcriptome and metabolome data, we can gain insights into the coordinated regulation of gene expression and metabolic pathways in response to salt stress. By plotting the KEGG pathways based on the transcriptome and metabolome data, we can elucidate the specific genes and metabolic pathways that are modulated under salt stress conditions. Our findings indicate that there are similarities between these two types of data. Specifically, we observed that the sucrose metabolism pathway is activated in the early stages of salt treatment (**Figure 7**), suggesting the involvement in the plant in response to salt stress. Sucrose, a disaccharide composed of glucose and fructose, serves as the predominant sugar in plant storage organs and represents the principal end product of photosynthetic activity. The compound under investigation is comprised of two monosaccharides, namely D-glucose and D-fructose (Keunen et al., 2013; Singh et al., 2016). The present study investigates the impact of salt stress on the sucrose composition of salt-tolerant genotype plants (**Figure 4D**). It was found that trehalose exhibits the ability to offer adequate permeation-protection and stabilize cell membranes as well as proteins (Paul et al., 2008). The presence of trehalose in plants is typically found in low concentrations, with its synthesis being primarily induced by abiotic stress factors (Kaplan and Guy, 2004; Panikulangara et al., 2004; Rizhsky et al., 2004; Guy et al., 2008). In rice, it has been observed that the overexpression of specific isoforms of TPS (trehalose-6-phosphate synthase) leads to enhanced resistance against adverse environmental conditions such as cold, drought, and salinity (Li et al., 2011). Meantime, cold stress leads to a transient increase in trehalose levels and TPP (trehalose-6-phosphate phosphatase) activity in rice (Panikulangara et al., 2004). Therefore, further exploration of the underlying molecular and biochemical processes involved in this sucrose enrichment could provide valuable insights into the mechanisms of sucrose enrichment in salt-resistant genotypes under salt stress has been identified as a plant defense mechanism (Yin et al., 2010).

## Conclusions

We have successfully constructed a comprehensive network of metabolites in tobacco plants in response to salt stress treatment. DMs revealed that disaccharides, heterocyclic compounds, monosaccharides, cholines, phenylacetic acids, amino acids and peptides, fatty acyl glycosides, eicosanoids, and fatty acids and conjugates were enriched in tobacco when exposure to salt stress. Integration of transcriptome and metabolome data has revealed the involvement of sucrose metabolism pathway in plants under salt stress conditions. By unraveling the intricate interplay between gene expression and metabolite levels, our study provides valuable insights into the molecular mechanisms underlying plant responses to salt stress. This integrative analysis serves as a foundation for further exploration and understanding of the complex regulatory networks governing plant stress responses.

## Data Availability

The datasets generated during the current study are available in the National Genomics Data Center (NGDC) database repository with accession PRJCA022888.
